# Low-dose aspirin use and survival in colorectal cancer: results from a population-based cohort study

**DOI:** 10.1186/s12885-018-4142-y

**Published:** 2018-02-27

**Authors:** Ronan T. Gray, Helen G. Coleman, Carmel Hughes, Liam J. Murray, Chris R. Cardwell

**Affiliations:** 1 Cancer Epidemiology and Health Services Research Group, Centre for Public Health, Queen’s University Belfast, Royal Victoria Hospital, Belfast, Northern Ireland BT12 6BA UK; 2 School of Pharmacy, Queen’s University Belfast, 97 Lisburn Road, Belfast, Northern Ireland BT9 7BL UK

**Keywords:** Aspirin, Colorectal cancer, Survival, Pharmaco-epidemiology

## Abstract

**Background:**

Aspirin has been proposed as a novel adjuvant agent in colorectal cancer (CRC). Six observational studies have reported CRC-specific survival outcomes in patients using aspirin after CRC diagnosis but the results from these studies have been conflicting. Using a population-based cohort design this study aimed to assess if low-dose aspirin use after diagnosis reduced CRC-specific mortality.

**Methods:**

A cohort of 8391 patients with Dukes’ A-C CRC (2009–2012) was identified from the Scottish Cancer Registry and linked to national prescribing and death records. Adjusted hazard ratios (HRs) and 95% confidence intervals (CIs) for CRC-specific mortality were calculated using time-dependent Cox regression.

**Results:**

There were 1064 CRC-specific deaths after a median follow-up of 3.6 years. Post-diagnostic low-dose aspirin use was not associated with a reduction in CRC-specific mortality either before or after adjustment for confounders (adjusted HR = 1.17, 95% CI 1.00–1.36). In sensitivity analysis pre-diagnostic low-dose aspirin was also not associated with reduced CRC-specific mortality (adjusted HR = 0.96, 95% CI 0.88–1.05).

**Conclusion:**

Low-dose aspirin use, either before or after diagnosis, did not prolong survival in this population-based CRC cohort.

## Background

Numerous observational studies and long term follow-up of randomised trials (of aspirin for cardiovascular indications) suggest that aspirin use is associated with a reduced risk of colorectal neoplasia [1]. Clinical trails have also confirmed that aspirin can reduce the risk of developing recurrent adenomatous polyps [2] in the general population and incident colorectal cancer (CRC) in Lynch syndrome carriers [3]. More recently, further evidence from long-term follow-up of these cardiovascular trials suggests aspirin could reduce metastases in CRC patients and therefore could have utility in CRC treatment [4]. To date, six epidemiological studies [5–10] have investigated the association between aspirin use after diagnosis and CRC-specific mortality. However, these studies reached different conclusions (two observed no association [5, 6] whilst four observed marked reductions [7–10]), and had various limitations including small sample size, [6] restriction to subgroups, [6] the potential for biased estimates (described later) [7, 8] and self-reported exposure ascertainment [9]. Although additional studies have investigated post-diagnostic aspirin use and all-cause mortality in CRC, [11] results of these studies could reflect non-cancer related events. Cancer-specific mortality limits events to those related to the cancer and represents a more specific endpoint for evaluating the efficacy of an adjuvant therapy [12].

Additional studies assessing aspirin use after diagnosis and CRC survival are therefore warranted to investigate the potential role of adjuvant aspirin treatment in CRC. We therefore investigated whether post-diagnostic aspirin use was associated with improved CRC-specific mortality in a Scottish population-based CRC cohort.

## Methods

### Data source

The study utilised linkages between national datasets from Scotland including the Scottish Cancer Registry (SMR06), the Prescribing Information System (available from January 2009 to January 2015), the General/Acute Inpatient and Day Case dataset (SMR01), the Outpatient Attendance dataset (SMR00) and the National Records of Scotland Death Records. Linkages between data sources were conducted using the Community Health Index number (unique to individuals in Scotland). The Privacy Advisory Committee of the National Health Service (NHS) National Services Scotland (NSS) approved the study.

### Study population

**Fig. 1:**
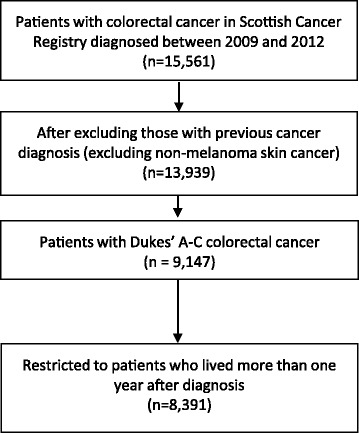
Patient selection for analysis in cohort study

A cohort of newly diagnosed Dukes’ A-C CRC patients was identified within the Scottish Cancer Registry (ICD codes of the colon C18 or rectum C20 including the recto-sigmoid junction C19) between January 2009 and December 2012. Cohort members with previous cancer diagnoses (after January 1999), apart from in situ neoplasms and non-melanoma skin cancers, were excluded (Fig. 1). Deaths were identified from National Records of Scotland with coverage up to 1st January 2015 (or from Scottish Cancer Registry death records) with CRC-specific deaths defined as those with underlying cause of death ICD code C18, C19, C20, C21 (anus) or C26 (other and ill-defined digestive organs). Deaths in the first year after CRC diagnosis were removed, this restriction reduces the likelihood of including patients who were not recurrence-free at exposure [13]. Patients were therefore followed up from 1 year after CRC diagnosis to death, the date they left Scotland or 1st January 2015.

### Exposure data

The Prescribing Information System (available from January 2009 to January 2015) holds all medicines dispensed in the community in Scotland [14]. Low-dose (75 mg) aspirin exposure was identified from dispensing records within this database. A quantity of 28 tablets was assumed for the less than 0.1% of prescriptions where quantity was assumed incorrect. Daily defined doses (DDD) were calculated based on the quantity of tablets as defined by the World Health Organisation [15]. Aspirin use was investigated as a time-varying covariate (patients were initially considered non-users and then users after a lag of 6 months after their first aspirin prescription) [13, 16]. This lag ensured new prescriptions in the six-month period prior to death were not considered as they may reflect end of life treatment. Dose (exposure)-response analyses were conducted with individuals considered non-users prior to 6 months after first use (i.e. aspirin users were considered non-users until the lag period passed), a short term user between 6 months after first use and 6 months after the 12th prescription (i.e. up to 1 year of exposure after the lag period) and a longer term user after this time (users for more than 1 year after the lag period).

### Covariates

Data available from the Scottish Cancer Registry included Dukes’ stage, histological grade and surgery, chemotherapy and radiotherapy in the 6 months after diagnosis. Comorbidities that contribute to the Charlson index were determined prior to diagnosis based upon ICD10 diagnosis codes, as described previously, [17] in Scottish hospital inpatient (SMR01) and outpatient data (SMR00). The SMR01 dataset contains information on hospital diagnoses and operations and the SMR00 dataset (both available from January 1999 to January 2015) contains diagnosis and procedures from new and follow up appointments at outpatient clinics. A deprivation measure was determined using the 2009 Scottish Index of Multiple Deprivation based upon postcode of residence [18].

### Statistical analysis

Time-dependent Cox regression models were used to calculate hazard ratios (HRs) for CRC-specific death and 95% confidence intervals (CIs) for aspirin users compared with non-users as described previously [19]. Multivariable analyses adjusted for the potential confounders age, sex, year of diagnosis, deprivation, site (colon or rectum), stage, grade, cancer treatment within 6 months (radiotherapy, chemotherapy, surgery), comorbidities (prior to diagnosis, including acute myocardial infarction, congestive heart failure, peripheral vascular disease, cerebral vascular accident, pulmonary disease, peptic ulcer, liver disease, diabetes, renal disease) and statin use (as a time-varying covariate). Analyses were conducted by number of tablets and repeated for all-cause mortality. Subgroup analyses were conducted by site (colon or rectal) and pre-diagnostic aspirin use (de novo versus pre- and post-diagnostic aspirin use).

Sensitivity analysis was conducted by increasing the lag period (the time from first dispensed aspirin prescription to when the patient started to accrue aspirin exposure follow-up time) to 1 year. A simplified analysis was also performed using Cox regression to assess survival in aspirin users compared to non-users in the first year after colorectal cancer diagnosis in individuals living more than 1 year after diagnosis (follow-up commenced 1 year from the date of diagnosis until the date of last data collection); this controls for immortal time bias without requiring time-varying covariates [20]. Finally, an analysis was conducted based upon aspirin prescriptions in the year prior to diagnosis (excluding patients diagnosed in 2009 for whom a full year of prescription records prior to diagnosis may not be available), not excluding deaths in the first year after diagnosis and including all colorectal cancer patients regardless of Dukes’ stage. The adjusted model for pre-diagnostic aspirin use did not include stage, grade or cancer treatment to avoid over adjustment [21, 22] as they could be on the causal pathway for the association between pre-diagnostic aspirin use and CRC-specific mortality.

An adjusted post-hoc analysis was undertaken comparing cardiovascular deaths between post-diagnostic aspirin users and non-users. Cardiovascular deaths were those in which the underlying death was ICD 10 codes I0–99, G45, Q20–26, F01 or equivalent ICD-9 codes. The adjusted model for this analysis was similar to the primary multivariable analysis except only non-cardiovascular comorbidities (pulmonary disease, peptic ulcer, liver disease, diabetes, renal disease) were included. All statistical analyses were conducted in STATA 13 software (StataCorp, College Station, TX).

## Results

A total of 8391 incident Dukes’ A to C CRC cases met the inclusion criteria (Fig. 1), in which there was on average 3.6 years of follow-up after diagnosis (median = 3.6, range1–6 years). Patient characteristics by aspirin use are shown in Table 1. Aspirin users in the year after diagnosis were more likely to be older, male and reside in more deprived areas. Stage and grade were generally similar by aspirin use, but a smaller proportion of aspirin users compared to non-users had Dukes’ C disease (post-diagnostic use, 32.7% versus 35.5% respectively). Aspirin users were also more likely to have comorbidities (particularly cardiovascular disease and diabetes) and use statins but a smaller proportion received adjuvant chemotherapy.

**Table 1:** Characteristics of colorectal cancer patients by post-diagnostic aspirin use

	Aspirin use in first year after cancer diagnosis^a^			
	Yes (*n* = 2150)		No (*n* = 6241)	
	Number	%	Number	%
Age				
< 50	13	0.6	432	6.9
50–59	131	6.1	1113	17.8
60–69	586	27.3	1973	31.6
70–79	934	43.4	1876	30.1
≥ 80	486	22.6	847	13.6
Gender				
Men	1387	64.5	3287	52.7
Deprivation (fifth)				
1 (most deprived)	447	20.8	1039	16.6
2	497	23.1	1272	20.4
3	429	20.0	1263	20.2
4	412	19.2	1361	21.8
5 (least deprived)	365	17.0	1305	20.9
Tumour location				
Colon	1493	69.4	4227	67.7
Rectum/rectosigmoid junction	657	30.6	2014	32.3
Dukes’ stage				
A	597	27.8	1683	27.0
B	851	39.6	2340	37.5
C	702	32.7	2218	35.5
Grade				
Well differentiated	82	3.8	203	3.3
Moderately differentiated	1646	76.6	4796	76.8
Poorly differentiated	315	14.7	968	15.5
Missing	107	5.0	274	4.4
Treatment (within six months)				
Surgery	2020	94.0	5908	94.7
Radiotherapy	192	8.9	679	10.9
Chemotherapy	472	22.0	2167	34.7
Comorbidity before cancer diagnosis				
Acute myocardial infarction	300	14.0	103	1.7
Congestive heart failure	135	6.3	94	1.5
Peripheral vascular disease	145	6.7	80	1.3
Cerebral vascular accident	162	7.5	141	2.3
Pulmonary disease	207	9.6	383	6.1
Peptic ulcer	59	2.7	151	2.4
Liver disease	6	0.3	25	0.4
Diabetes	290	13.5	285	4.6
Renal disease	87	4.0	102	1.6
Statin use after diagnosis	1647	76.6	1490	23.9

^a^Restricted to patients who survived at least 1 year after diagnosis

Post-diagnostic aspirin use was not associated with reduced CRC-specific mortality after adjustment for potential confounders (HR = 1.17, 95% CI 1.00–1.36) (Table 2). Results for overall survival were similar, except that the observed increases in mortality were detected as statistically significant after adjustment (adjusted HR = 1.21 95%CI 1.07–1.37). *Post-hoc* analysis confirmed there was a marked increase in cardiovascular deaths in post-diagnostic aspirin users compared to non-users (adjusted HR = 1.63, 95% CI 1.15–2.29).

**Table 2:** Association between aspirin use after diagnosis and CRC-specific and overall survival in patients with colorectal cancer

	Mortality	Patients	Person years	Unadjusted HR (95% CI)	*P* value	Adjusted^a^ HR (95% CI)	*P* value
CRC-specific survival							
Aspirin non-user	729	5881	15,957	1.00 (ref. cat.)		1.00 (ref. cat.)	
Aspirin user	335	2510	6178	1.22 (1.07, 1.39)	0.003	1.17 (1.00, 1.36)	0.06
1 to 365 DDDs vs. non-user	160	725	2549	1.34 (1.12, 1.59)	0.001	1.23 (1.02, 1.50)	0.03
≥ 365 DDDs vs. non-user	175	1785	3629	1.12 (0.95, 1.33)	0.18	1.10 (0.90, 1.33)	0.36
Overall survival							
Aspirin non-user	1035	5881	15,957	1.00 (ref. cat.)		1.00 (ref. cat.)	
Aspirin user	600	2510	6178	1.51 (1.37, 1.67)	< 0.001	1.21 (1.07, 1.37)	0.002
1 to 365 DDDs vs. non-user	263	725	2549	1.59 (1.39, 1.83)	< 0.001	1.26 (1.08, 1.46)	0.004
≥ 365 DDDs vs. non-user	337	1785	3629	1.45 (1.28, 1.65)	< 0.001	1.18 (1.02, 1.37)	0.03

*Abbreviations*: *CI* confidence interval, *CRC* colorectal cancer, *DDD* daily defined dose, *HR* hazard ratio

^a^Multivariable model contains age, sex, year of diagnosis, deprivation, site (colon or rectum), stage, grade, cancer treatment within 6 months (radiotherapy, chemotherapy, surgery), comorbidities (prior to diagnosis, including acute myocardial infarction, congestive heart failure, peripheral vascular disease, cerebral vascular accident, pulmonary disease, peptic ulcer, liver disease, diabetes, renal disease) and statin use (as a time-varying covariate)

There was no evidence of an exposure response when users consuming more (long term users) or less (short term users) than a 365-day supply were compared with non-users (Table 2). Stratifying by tumour location did not greatly alter the size or direction of the association for CRC-specific mortality (adjusted HR = 1.13, 95% CI 0.94–1.36 versus adjusted HR = 1.26, 95% CI 0.94–1.69 in colon and rectal cancer patients respectively).

The results of the planned sensitivity analyses are documented in Tables 3 and 4. Repeating the analysis using a simplified one-year analysis did not demonstrate a survival benefit for post-diagnostic aspirin use. Interestingly cancer-specific and overall survival were markedly worse in de novo post-diagnostic aspirin users (adjusted CRC-specific survival HR = 1.51, 95% CI 1.12–2.05; adjusted overall survival HR = 1.53, 95% CI 1.19–1.95) when post-diagnostic aspirin use versus non-use was stratified by pre-diagnostic aspirin use. However, when aspirin use was compared to non-use in the year prior to diagnosis, there was no association with CRC-specific (adjusted HR = 0.96, 95% CI 0.88–1.05) or overall survival (adjusted HR = 0.99, 95% CI 0.92–1.06). Finally, the results of the main time-varying analysis of post-diagnostic aspirin use were not greatly altered when the lag period was increased from 6 to 12 months (adjusted cancer-specific survival HR = 1.08, 95%CI 0.94–1.26; adjusted overall survival HR = 1.15, 95% CI 1.03 to 1.29).

**Table 3:** Sensitivity analyses for the association between aspirin use and CRC-specific survival in patients with colorectal cancer

	Aspirin user			Aspirin non-user						
	Mortality	Patients	Person years	Mortality	Patients	Person years	Unadjusted HR (95% CI)	*P* value	Adjusted HR (95% CI)	*P* value
CRC-specific survival										
Post-diagnostic aspirin use (fixed time exposure in first year)^a^										
Aspirin user vs. non-user	310	2150	5692	754	6241	16,442	1.19 (1.05, 1.36)	0.01	1.10 (0.94, 1.29)	0.22
Post-diagnostic aspirin use versus non-use stratified by pre-diagnostic use (excludes 2009 cases)^b^										
Pre-diagnostic users	168	1333	2983	46	257	614	0.75 (0.54,1.04)	0.08	0.80 (0.56,1.13)	0.20
Pre-diagnostic non-users	51	435	744	467	4301	9930	1.52 (1.13,2.03)	0.01	1.51 (1.12,2.05)	0.01
Aspirin use vs. non-use in year before colorectal cancer diagnosis (excludes 2009 cases)^c^										
Aspirin user vs. non-user	1104	2853	6460	2551	7555	18,652	1.23 (1.14, 1.32)	< 0.001	0.96 (0.88, 1.05)	0.37

*Abbreviations*: *CI* confidence interval, *CRC* colorectal cancer, *HR* hazard ratio

^a^Model contains age, sex, year of diagnosis, deprivation, site (colon or rectum), stage, grade, cancer treatment within six months (radiotherapy, chemotherapy, surgery), comorbidities (prior to diagnosis, including acute myocardial infarction, congestive heart failure, peripheral vascular disease, cerebral vascular accident, pulmonary disease, peptic ulcer, liver disease, diabetes, renal disease) and statin use (in first year after diagnosis)

^b^Excluding patients diagnosed in 2009 (who do not have complete prescription records for year before diagnosis). Based upon main time-varying covariate multivariable model containing age, sex, year of diagnosis, deprivation, site (colon or rectum), stage, grade, cancer treatment within 6 months (radiotherapy, chemotherapy, surgery), comorbidities (prior to diagnosis, including acute myocardial infarction, congestive heart failure, peripheral vascular disease, cerebral vascular accident, pulmonary disease, peptic ulcer, liver disease, diabetes, renal disease) and statin use (as time-varying covariate)

^c^Excluding patients diagnosed in 2009 (who do not have complete prescription records for year before diagnosis) but not excluding patients who die within 1 year of diagnosis; adjusted model contains age, sex, year of diagnosis, deprivation, site (colon or rectum), comorbidities (prior to diagnosis, including acute myocardial infarction, congestive heart failure, peripheral vascular disease, cerebral vascular accident, pulmonary disease, peptic ulcer, liver disease, diabetes, renal disease) and statin use (in year prior to diagnosis)

**Table 4:** Sensitivity analyses for the association between aspirin use and overall survival in patients with colorectal cancer

	Aspirin user			Aspirin non-user						
	Mortality	Patients	Person years	Mortality	Patients	Person years	Unadjusted HR (95% CI)	*P* value	Adjusted HR (95% CI)	*P* value
Overall survival										
Post-diagnostic aspirin use (fixed time exposure in first year)^a^										
Aspirin user vs. non-user	554	2150	5692	1081	6241	16,442	1.48 (1.33, 1.64)	< 0.001	1.17 (1.03, 1.32)	0.01
Post-diagnostic aspirin use versus non-use stratified by pre-diagnostic use (excludes 2009 cases)^b^										
Pre-diagnostic users	290	1333	2983	70	257	614	0.84 (0.65,1.10)	0.21	0.88 (0.67,1.17)	0.39
Pre-diagnostic non-users	84	435	744	638	4301	9930	1.79 (1.42,2.25)	< 0.001	1.53 (1.19,1.95)	0.001
Aspirin use vs. non-use in year before colorectal cancer diagnosis (excludes 2009 cases)^c^										
Aspirin user vs. non-user	1506	2853	6460	3117	7555	18,652	1.37 (1.29, 1.46)	< 0.001	0.99 (0.92, 1.06)	0.77

*Abbreviations*: *CI* confidence interval, *HR* hazard ratio

^a^Model contains age, sex, year of diagnosis, deprivation, site (colon or rectum), stage, grade, cancer treatment within six months (radiotherapy, chemotherapy, surgery), comorbidities (prior to diagnosis, including acute myocardial infarction, congestive heart failure, peripheral vascular disease, cerebral vascular accident, pulmonary disease, peptic ulcer, liver disease, diabetes, renal disease) and statin use (in first year after diagnosis)

^b^Excluding patients diagnosed in 2009 (who do not have complete prescription records for year before diagnosis). Based upon main time-varying covariate multivariable model containing age, sex, year of diagnosis, deprivation, site (colon or rectum), stage, grade, cancer treatment within 6 months (radiotherapy, chemotherapy, surgery), comorbidities (prior to diagnosis, including acute myocardial infarction, congestive heart failure, peripheral vascular disease, cerebral vascular accident, pulmonary disease, peptic ulcer, liver disease, diabetes, renal disease) and statin use (as time-varying covariate)

^c^Excluding patients diagnosed in 2009 (who do not have complete prescription records for year before diagnosis) but not excluding patients who die within 1 year of diagnosis; adjusted model contains age, sex, year of diagnosis, deprivation, site (colon or rectum), comorbidities (prior to diagnosis, including acute myocardial infarction, congestive heart failure, peripheral vascular disease, cerebral vascular accident, pulmonary disease, peptic ulcer, liver disease, diabetes, renal disease) and statin use (in year prior to diagnosis)

## Discussion

Low-dose aspirin use after colorectal cancer diagnosis was not associated with improved CRC-specific survival in this large Scottish population-based cohort study. Specifically, low-dose aspirin use was associated with a 17% increase in CRC-specific mortality, but this was not statistically significant and did not follow an exposure response. Overall, in subgroup and sensitivity analyses the findings of the main analyses were relatively consistent.

Six observational studies have reported the association between CRC-specific outcomes and post-diagnostic aspirin use in CRC [5–10]. The findings from the present study add further inconsistency to the evidence base therefore the differences between the studies should be discussed. These findings are within the confidence intervals for the null association our research group previously reported using linked data from the Clinical Practice Research Datalink and the English National Cancer Data Repository (*n* = 4794, adjusted CRC-specific survival HR = 0.99, 95% CI 0.86–1.15) [5]. Kothari et al. also reported a null association between CRC-specific mortality and low-dose aspirin use at CRC diagnosis, but the study was small and limited to tumours that were PIK3CA mutant (*n* = 185) [6].

Notably, our current results do contrast with the inverse association reported by McCowan et al. (2013) in a separate regional Scottish population-based study [8]. Important differences in the statistical analysis could explain these findings. In particular, their analysis used an unlagged start/stop time-varying covariate. This potentially introduces bias through reverse causation as deaths were allocated to the exposed/unexposed group on the basis of whether the aspirin prescription covered the death date [5, 23]. In particular, prescription medications may be stopped as patients enter the terminal phase of their disease [24]. Failure to include a lag period can therefore over estimate any association or inadvertently make it appear that aspirin use is associated with a reduced risk of disease progression due to misclassification.

Similarly, Goh et al. (2014) reported a 62% reduction (HR = 0.38, 95% CI 0.17–0.84) in CRC-specific mortality among *n* = 726 post-diagnostic aspirin users utilising a national prescription database [7]. However the authors do not describe any method to eliminate immortal time bias (such as use of a time-varying covariate) which could artificially create a protective effect [16]. It is also worth noting that our study only assessed low-dose aspirin use, therefore we could not investigate the improved outcomes for predominantly high-dose aspirin users observed in previous studies [9]. Finally, despite using broadly similar methodology in large population-based CRC cohorts, the results of the current study conflict with the modest protective association (adjusted HR = 0.85 95% CI 0.79, 0.92) observed with three or more aspirin prescriptions reported by Bains et al. [10]. It is unclear why the results of these studies differ but some of this could reflect differences in the confounding variables that were included in multivariable analyses; in particular, only the current study adjusted for chemotherapy or radiotherapy use, the presence of comorbid illnesses and deprivation.

The 20% increase in all-cause mortality in aspirin users compared to non-users in this colorectal cancer cohort warrants further discussion but as post hoc analysis demonstrated that this increase was driven by cardiovascular mortality, it seems likely that confounding by indication is responsible. Residual confounding may also bias the CRC-specific analyses reported in this study. In particular, the prevalence of cardiovascular disease in Scotland is amongst the highest in Europe and is the highest of all the regions in the UK [25]. Cardiovascular disease is known to be associated with reduced physical activity and obesity, two factors that are independently associated with worse survival outcomes in colorectal cancer [26–28]. Unfortunately it was not possible to adjust for these lifestyle variables in the current cohort. Therefore, as low-dose aspirin is prescribed for the secondary prevention of cardiovascular disease, obesity and physical inactivity may be associated with both the exposure and the outcome of interest.

The strengths of the current study include its size, population-based design and use of dispensing information with detailed information. The contemporary nature of the cohort and its population-based approach also enhance the external validity of the results. One of the main limitations is that as this is an observational study there is the potential for residual confounding. In addition to the absence of information on lifestyle variables, the comorbidity data may not be complete as it relied on hospital records rather than GP recorded diagnoses or patient interviews. Also, while dispensing information is more robust than prescribing information, compliance cannot be confirmed. Over-the-counter usage could also result in some users being misclassified but a previous UK study using GP prescribing data estimated that 70–80% [29] of aspirin use in the age-group we investigated was prescription-based, while another showed little evidence of misclassification by aspirin usage when compared with patient recall [30]. Cause of death can also be misclassified when relying on data from national statistics records. We were also unable to assess cyclooxygenase 2 (COX-2) expression or PIK3CA mutation status, two potential molecular biomarkers that may predict response to aspirin therapy [9, 31, 32]. Importantly though, while these markers did differentiate tumours more likely to benefit from aspirin use in the these studies, the survival benefit associated with aspirin use also existed before stratification of the cohorts by molecular profile [9, 32]. Finally, follow-up in the current cohort is relatively short (median = 3.6 years). However, in a separate Scottish cohort study, McCowan et al. demonstrated a survival benefit associated with aspirin use after a shorter median follow-up of 2.8 years [8].

## Conclusion

In summary, there is inconsistent evidence from previous observational studies on the association between post-diagnostic low-dose aspirin use and improved CRC-specific survival. In the present study we did not find any evidence of a reduction in cancer-specific mortality in aspirin users. Although we observed a small increase in all-cause mortality this seems likely to reflect confounding by indication. For these reasons clinical trials assessing adjuvant aspirin therapy in colorectal cancer (NCT02467582; NCT02301286; NCT00565708; ISRCTN74358648) are keenly anticipated but are not due to report until at least 2022.

## Abbreviations

CI: Confidence interval
CRC: Colorectal cancer
DDD: Daily defined dose
HR: Hazard ratio
